# Special Issue on “Raman Spectroscopy for Chemical and Structural Characterization in Biology”

**DOI:** 10.3390/ijms231911795

**Published:** 2022-10-04

**Authors:** Sébastien Bonhommeau

**Affiliations:** Institut des Sciences Moléculaires, UMR 5255, University of Bordeaux, CNRS, Bordeaux INP, F-33400 Talence, France; sebastien.bonhommeau@u-bordeaux.fr

Raman spectroscopy is a popular non-invasive spectroscopic technique for molecular characterization and imaging with a high spatial resolution. In biology, it is particularly appropriate for the identification of biomolecules and the specific spectral signatures of cells [1]. In this Special Issue, six experimental studies and a review explore the performance of Raman spectroscopy to probe animal [2,3,4,5,6], vegetal [7], and fungal [8] cells, obtaining original information via data processing using multivariate analysis methods such as principal component analysis (PCA) [3,5,8], hierarchical cluster analysis (HCA) [3,7], and multiple curve resolution (MCR) [7] approaches.

Confocal Raman microscopy and surface-enhanced Raman spectroscopy (SERS) present fascinating applications in cancer nanomedicine [2]. These techniques allow for the bioimaging of various cancer cells (e.g., breast, prostate, and colorectal cancers; sarcoma; and carcinoma) and for the detection of different biomarkers (e.g., exosomes) in order to improve cancer diagnostics and therapy in a strategy to develop cancer nanotheranostics. Moreover, despite a comparable biochemical background, fine differences are observed in the Raman signatures of diverse bone marrow mesenchymal stem/stromal cell populations [3]. This suggests possible advances to better understand inter-individual stem cell diversity, which may have a potential impact on the development of personalized therapies. Visible Raman microscopic imaging is also used to examine the cellular response to the bleomycin anticancer drug-generating single- and double-strand breaks in the DNA of living cells [4]. It reveals some of the conformational transition from B-DNA to A-DNA as well as the increased expression of proteins within the cell nucleus. In addition, RNA modifications in human sperm, such as the methylation of the RNAs adenine and guanine, can be probed by means of UV resonance Raman spectroscopy [5]. This could facilitate the selection of high-quality sperm samples for assisted reproductive technologies.

Thanks to its high spatial resolution of a few hundreds of nanometers, Raman spectroscopy is an ideal label-free tool to characterize single cells [4,7]. Confocal Raman microscopy allows single birch pollen grains to be distinguished from each other depending on their collection site due to differences in their sporopollenin, lipid, and protein composition [7]. It also reveals chemical groups reflecting the occurrence of DNA repair processes in living cells [4] and the presence of ancient organic residues (e.g., proteins and nucleic acids) in fossilized bone tissues [6]. Finally, Fourier transform Raman spectroscopy can be employed to identify chemical biomass constituents of Mucoromycota filamentous fungal strains (i.e., lipids, proteins, carbohydrates, polyphosphates, and carotenoids) and is able to lead to quantitative information on the lipid, phosphate, and carotenoid content provided that convenient data processing is performed [8].

As a whole, this Special Issue covers various practical, technical and applicative aspects of Raman spectroscopy for analytical (mainly medical/clinical) purposes. It provides significant contributions for which I sincerely congratulate the authors.
